# MSIFinder: a python package for detecting MSI status using random forest classifier

**DOI:** 10.1186/s12859-021-03986-z

**Published:** 2021-04-12

**Authors:** Tao Zhou, Libin Chen, Jing Guo, Mengmeng Zhang, Yanrui Zhang, Shanbo Cao, Feng Lou, Haijun Wang

**Affiliations:** 1 AcornMed Biotechnology Co., Ltd., Floor 18, Block 5, Yard 18, Kechuang 13 RD, Beijing, 100176 China; 2 Department of Pathology, The Second Affiliated Hospital of Zhejiang University School of Medicine, No. 88 Jiefang Road, Shangcheng District, Hangzhou, 310009 Zhejiang China

**Keywords:** Microsatellite instability, Genome sequencing, Machine learning technology, Random forest classifier, Immunotherapy

## Abstract

**Background:**

Microsatellite instability (MSI) is a common genomic alteration in colorectal cancer, endometrial carcinoma, and other solid tumors. MSI is characterized by a high degree of polymorphism in microsatellite lengths owing to the deficiency in the mismatch repair system. Based on the degree, MSI can be classified as microsatellite instability-high (MSI-H) and microsatellite stable (MSS). MSI is a predictive biomarker for immunotherapy efficacy in advanced/metastatic solid tumors, especially in colorectal cancer patients. Several computational approaches based on target panel sequencing data have been used to detect MSI; however, they are considerably affected by the sequencing depth and panel size.

**Results:**

We developed MSIFinder, a python package for automatic MSI classification, using random forest classifier (RFC)-based genome sequencing, which is a machine learning technology. We included 19 MSI-H and 25 MSS samples as training sets. First, we selected 54 feature markers from the training sets, built an RFC model, and validated the classifier using a test set comprising 21 MSI-H and 379 MSS samples. With this test set, MSIFinder achieved a sensitivity (recall) of 1.0, a specificity of 0.997, an accuracy of 0.998, a positive predictive value of 0.954, an F1 score of 0.977, and an area under the curve of 0.999. To further verify the robustness and effectiveness of the model, we used a prospective cohort consisting of 18 MSI-H samples and 122 MSS samples. MSIFinder achieved a sensitivity (recall) of 1.0 and a specificity of 1.0. We discovered that MSIFinder is less affected by a low sequencing depth and can achieve a concordance of 0.993 while exhibiting a sequencing depth of 100×. Furthermore, we realized that MSIFinder is less affected by the panel size and can achieve a concordance of 0.99 when the panel size is 0.5 M (million bases).

**Conclusion:**

These results indicate that MSIFinder is a robust and effective MSI classification tool that can provide reliable MSI detection for scientific and clinical purposes.

**Supplementary Information:**

The online version contains supplementary material available at 10.1186/s12859-021-03986-z.

## Background

Microsatellites (MS), also known as short tandem repeats, are tandemly repeated sequences with typical repeat unit lengths ranging from 1 to 6 bases in genome sequences. When the mismatch repair (MMR) system has a deficiency, these spontaneous mutations in microsatellites cannot be corrected. Therefore, they accumulate, causing the microsatellite sequence length or the base composition to change with the increase in tumor mutation burden. We define this process as microsatellite instability (MSI) [1]. MSI can promote carcinogenesis and play a major role in the mechanism of malignant transformation by favoring the accumulation of thousands of mutations in a broad spectrum of different anatomic sites such as colon, stomach, prostate, esophagus, endometrium, lung, and head and neck [2]. In addition, it has been demonstrated that MSI-H cancers are biologically marked by genomic instability, high mutation burden, and numbers of neoantigens and tumor-infiltrating lymphocytes (TILs), which makes MSI contribute to cancer immunology and useful for predicting the response to immunotherapy [3].

MSI was first discovered in colorectal cancer (CRC) in 1993 [4]. The detection of MSI has been proposed as a screening method for Lynch syndrome, stage II CRC prognostic factor, stage II CRC predictor factor of adjuvant chemotherapy, and advanced solid tumor predictive factor for immunotherapy efficacy [5, 6]. With the vigorous development of clinical research on immunotherapy, the listing of immunological checkpoint inhibitors, and the expansion of indications in the field of cancer, MSI is a predictive biomarker for the efficacy of immunotherapy in advanced/metastatic solid tumors, especially its detection is becoming increasingly important in colorectal cancer (CRC) patients.

Current MSI detection methods are as follows: (1) Analysis of MMR protein expression by IHC. Because MSI is generally caused by MMR protein deficiency, it can reflect the MSI status. The MMR system contains four MMR proteins, namely MLH1, MSH2, MSH6, and PMS2 [7]. Therefore, when there is a loss of one or more MMR protein expression, it is judged as dMMR. This loss of MMR protein expression observed via IHC has proven to be highly concordant with DNA-based MSI testing with good sensitivity (> 90%) and excellent specificity (100%) [8], with the premise being that the IHC detection platform is reliable and certified. However, many IHC detection platforms are unreliable, and the results of IHC detection platforms are not certified.

(2) Fluorescent multiplex polymerase chain reaction (PCR) assay for the identification of MSI [9–11]. The clinical diagnosis of MSI is usually achieved by examining the lengths of the PCR products of five informative microsatellite loci, which is the “gold standard” for detecting MSI. The National Cancer Institute (NCI) proposed the Bethesda/NCI panel for detecting MSI via two mononucleotide (BAT-25 and BAT-26) and three dinucleotide (D2S123, D5S346, and D17S250) repeat microsatellites [12]. Subsequently, a set of five quasi-monomorphic mononucleotide repeat microsatellites (BAT-25, BAT-26, NR-21, NR-22, and NR-24) were recommended [13], based on which tumors that present two or more unstable markers (or ≥ 30–40% if more markers are tested) should be defined as MSI/MSI-H. The other tumors are classified as microsatellite stable (MSS) or MSI-low (MSI-L) if no markers or only one marker is unstable (if more markers are tested, < 30–40% are unstable). However, this method has a low-throughput and needs to match the normal sample, making it less cost-effective.

(3) Computational methods for detecting MSI status in cancer. Since 2015, with the large-scale development of precision medicine, next-generation sequencing (NGS) has increased rapidly and has been widely used in clinical practice. Further, MSI algorithms are continuously being developed, and thus far, several software packages, such as mSINGS [13], MSIsensor [14], MSIseq [15], and MSIpred [16], have been able to accurately detect MSI. These software packages are mainly based on the changes in the length, mutation type, and mutation burden of microsatellite locus repeats during the detection of MSI. During the analysis phase, the software packages select markers that can distinguish between MSI-H and MSS and subsequently select a valid classification model to maximize the discrimination between MSI-H and MSS states. However, there are varying degrees of disadvantages with these software packages. For instance, mSINGS cannot select all the effective sites according to different panels; MSIsensor requires matched normal samples, thereby increasing costs; and MSIseq and MSIpred require a large panel size suitable for whole-exon sequencing.

We propose a software package that can detect the MSI status across multiple tumor types with high accuracy, sensitivity, and specificity and is not affected by the panel size and sequencing depth.

## Implementation

### Material

The training set included 19 MSI-H and 25 MSS samples. The test set included 21 MSI-H and 379 MSS samples. The prospective cohort included 18 MSI-H and 122 MSS samples. We collected 30 white blood samples from 30 patients, for selecting markers. Informed consent was obtained from all participants, and the study was approved by the Ethical Committee of the Second Affiliated Hospital of Zhejiang University School of Medicine. The libraries of all the samples were enriched using Xiangyi™ 808 cancer-gene panel (Acornmed Biotechnology Co., Ltd.), which encompassed 808 cancer-related genes and targeted genomes > 2.0 Mb. The PCR and fragment analysis of the paired normal and tumor tissue of the training set and the test set determined microsatellite instability (MSI) at the standard five NCI-recommended sites.

### Implementation

**Fig. 1:**
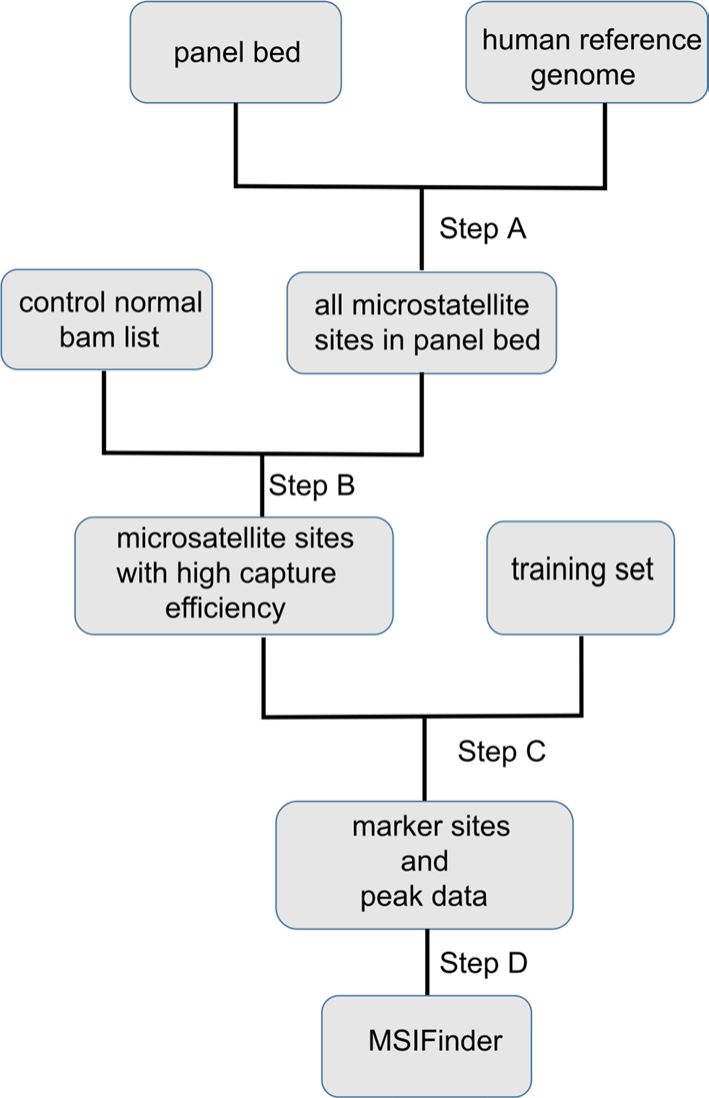
Flowchart for developing MSIFinder using random forest classifier

MSIFinder was written and tested using Python 3.51 and is freely available as a Python package. It requires pandas (version 0.23.4), pysam (version 0.15.1), and sklearn (version 0.20.0) packages to function properly. It also requires two external programs, MSIsensor (version 0.6) and bedtools (version 2.28.0). The workflow for developing MSIFinder is presented in Fig. 1. Step A: use MSIsensor and bedtools to scan microsatellites from a human reference genome (hg19/GRCh37) and then obtain all microsatellite sites in the panel bed (see “Select markers” section); Step B: calculate the average depth for all microsatellite sites and select the sites with high capture efficiencies using control samples (see “Select markers” section); Step C: obtain the final microsatellite sites by using the test set to determine the microsatellites with high capture efficiencies (see “Select markers” and “Definition of peak data” sections); Step D: use a random forest classifier (RFC) to build a machine learning classifier using the final microsatellite sites and peak data (see “Built and applied machine learning classifier” section).

### Data preprocessing

To obtain clean reads, quality control and preprocessing of FASTQ files from tumor tissue and white blood samples were done by fastp (version 0.19.3). Next, the Burrows–Wheeler aligner (BWA) (version 0.7.12-r1039) and SAMtools (version 0.1.19-96b5f2294a) were used to map the clean reads against the human reference genome (hg19/GRCh37) and perform alignment processing. Subsequently, sample-level, fully local indel realignment was performed using the genomic analysis toolkit (GATK) (version 4.1.0.0) and duplicate reads removed using Picard (version 1.72). The quality score was recalibrated using GATK to generate the final binary SAM (BAM) files used for subsequent analyses.

### Select markers

Here, MSIsensor (version 0.6) was used to scan microsatellites from the reference genome, with the minimal homopolymer size set to 10 and the reference genome sequence file set at hg19. Next, using the intersect mode of bedtools, the intersection with the panel bed was obtained. The sites with more than three repeats were chosen as candidate microsatellite sites.

(1) The depth of 30 normal control samples of the candidate microsatellite sites and the average depth was calculated. (2) The training set was analyzed, which comprised 19 MSI-H and 25 MSS samples with an average depth file to obtain the training data results. (3) The training data results from the sites with “Average_Total_Reads” greater than 30 were analyzed, and “Average_Number_Peak” greater than 1.5 were chosen as new candidate microsatellite sites. The Wilcoxon rank-sum test was used to compare the peak between the 19 MSI-H and 25 MSS samples, and the sites with a *p* value lesser than 0.01 were chosen as the final microsatellite sites.

### Built and applied machine learning classifier

We then developed two pan-tumor models, the RFC model and Support Vector Machines (SVM) model, with sklearn (version 0.20.0) for MSI classification using the aforementioned 54 markers of all the tumors from the training set. The random forest algorithm is not significantly affected by data with various dimensions and can handle a large number of dimensions, and it is more suitable for biological data [17]. SVMs are one of the most widely used and robust classifiers. We chose RFC by comparing its performance with that of SVM on the test set, including sensitivity, specificity, accuracy, PPV, and F1 score. For detailed results, see Additional file 1: Tables S1. For a new tumor tissue sample, MSIFinder identifies its peak data and offers the prediction score. If the score is greater than or equal to 0.6, the sample is termed MSI-H; otherwise, it is termed MSS.

### Comparison of MSIFinder with other software

Among previously published software tools, mSINGS and MSIsensor are similar to MSIFinder because they also use the number of repeats of different lengths present within each of the identified microsatellite sites as markers. Therefore, in this study, we compared the performances of mSINGS and MSIsensor with that of MSIFinder. The testing set of 400 samples utilized to validate the performance of MSIFinder was also used to evaluate mSINGS and MSIsensor. In this study, the parameters used for mSINGS and MSIsensor are the same as those used in [14, 18].

### Definition of peak data

**Fig. 2:**
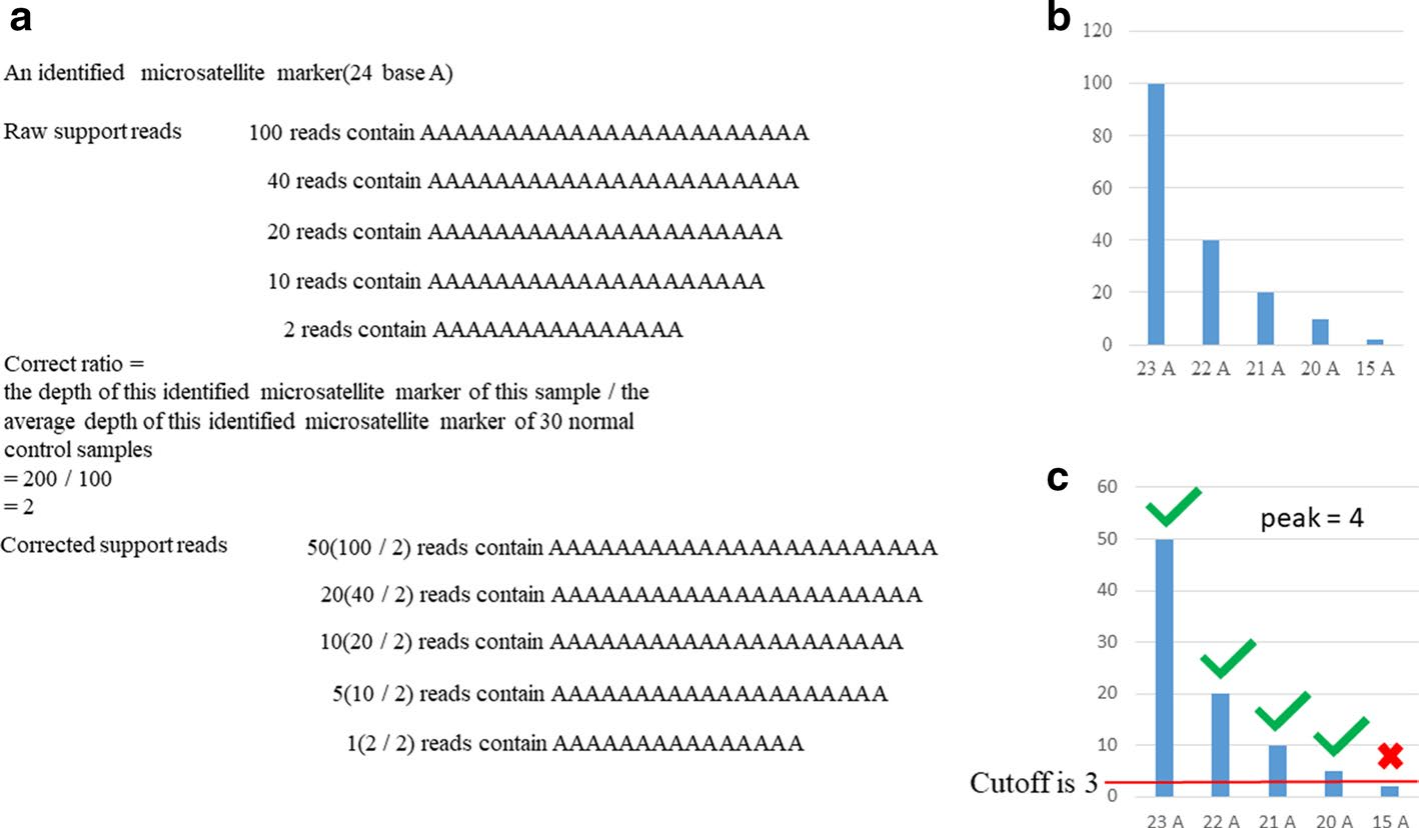
Peak calculation. **a** Obtaining the corrected support reads of an identified microsatellite marker consisting of 24 bases A. **b** The vertical coordinate shows the number of raw support reads. **c** The vertical coordinate shows the number of corrected support reads and the calculated peak

Peak data are the number of repeats of different lengths present within each of the identified microsatellite markers. To be more specific, if the corrected support reads are greater than 3, then the repeats are valid. For instance, if an identified microsatellite marker is 24 base A, the raw support reads of all the repeats are 2 support reads for 15 base A, 10 support reads for 20 base A, 20 support reads for 21 base A, 40 support reads for 22 base A, and 100 support reads for 23 base A. Because the sequencing depth affects the number of repeats of different lengths, we used the average depth of the identified microsatellite markers of 30 normal control samples to correct the support reads of all repeats. From the example above, the average depth of this identified microsatellite marker is 100×, and the sample sequence depth is 200×; therefore, the correct ratio is 2. The support reads of all repeats transformed are 1 support reads for 15 A, 5 support reads for 20 A, 10 support reads for 21 A, 20 support reads for 22 A, and 50 support reads for 23 A. Therefore, the number of repeats of different lengths of the identified microsatellite marker is 4 (Fig. 2), which is called the peak, and all the number of repeats of different lengths of identified microsatellite markers of one sample are called peak data.

### Computing resources

We used the rank sums function of scipy (version 1.1.0) in Python (version 3.5) to perform a Wilcoxon rank-sum test. We also used the chi-square function of scipy (version 1.1.0) to execute a chi-square test. The figures were generated using Matplotlib (version 3.03) and Seaborn (version 0.9.0) in Python (version 3.5).

## Results

### Built machine learning classifier

MSIFinder uses machine learning to perform MSI detection. The number of repeats of different lengths present within each of the identified microsatellite markers, defined as the peak data, is used as the feature data. Before classifying the MSI status of the tumor samples, a classifier that can distinguish between MSS and MSI-H needs to be constructed. First, we need to determine the microsatellite loci features in the classifier. This step is to maximize the discrimination between the positive and negative samples, i.e., feature selection. Based on the reference genome hg19, all 1435 microsatellite loci of the panel were obtained, and their sequencing depths were analyzed based on the white blood samples of 30 patients. 1328 loci with sequencing depth greater than 30 and average peak greater than 1.5 were selected as captured high-efficiency sites. The peak data for the high-capacity sites were calculated based on a training set comprising 19 MSI-H and 25 MSS samples. Finally, we obtained 54 loci with *p* value < 0.01, derived with a Wilcoxon rank-sum test, which included BAT-25, BAT-26, NR-21, NR-22, and NR-24 from NCI [13]. Table 1 shows detailed loci information of 54 microsatellites. Using these 54 microsatellite loci, we chose RFC to develop the classifier in the training set to perform a 10-time cross-validation receiver operating curve (ROC) analysis. When the predicted score is ≥ 0.6, the sample is MSI-H; otherwise, it is MSS. We referred to this classifier and the python pipeline calculating the peak data as MSIFinder. Additional file 2: Fig. S1 shows the performance of MSIFinder in the training set with an AUC of 1.0. For detailed results, see Additional file 1: Table S1.

**Table 1:** Composition of the 54 microsatellite loci

MSID	Chr	Start	End	MS[repeat]	MSID	Chr	Start	End	MS[repeat]
MS95	chr2	29,523,421	29,523,440	A[20]	MS583	chr7	140,482,264	140,482,279	A[16]
MS101	chr2	29,527,360	29,527,377	A[18]	MS598	chr7	140,496,149	140,496,164	A[16]
MS125	chr2	42,481,758	42,481,772	T[15]	MS603	chr7	140,498,360	140,498,380	T[21]
MS154	chr2	42,557,760	42,557,775	T[16]	MS701	chr8	38,281,181	38,281,201	A[21]
MS165	chr2	47,641,560	47,641,586	A[27]	MS752	chr9	133,712,212	133,712,233	A[22]
MS171	chr2	48,033,891	48,033,908	T[18]	MS766	chr9	133,721,247	133,721,259	A[13]
MS177	chr2	95,849,362	95,849,384	T[23]	MS767	chr9	133,721,469	133,721,496	TG[14]
MS210	chr2	215,593,006	215,593,025	A[20]	MS780	chr9	133,728,558	133,728,580	T[23]
MS211	chr2	215,593,262	215,593,276	T[15]	MS790	chr10	8,115,669	8,115,686	A[18]
MS228	chr3	12,633,425	12,633,440	T[16]	MS793	chr10	32,315,464	32,315,475	T[12]
MS230	chr3	12,634,231	12,634,252	T[22]	MS798	chr10	43,595,837	43,595,850	T[14]
MS233	chr3	12,635,286	12,635,304	T[19]	MS875	chr10	89,728,672	89,728,692	A[21]
MS237	chr3	12,639,510	12,639,524	T[15]	MS913	chr10	123,336,649	123,336,673	A[25]
MS245	chr3	12,656,094	12,656,105	T[12]	MS921	chr10	123,341,276	123,341,300	A[25]
MS309	chr3	185,787,291	185,787,309	T[19]	MS974	chr11	102,193,509	102,193,534	A[26]
MS311	chr3	185,787,763	185,787,772	T[10]	MS976	chr11	108,114,662	108,114,676	T[15]
MS331	chr4	25,680,310	25,680,328	T[19]	MS983	chr11	108,195,977	108,195,995	T[19]
MS340	chr4	55,598,212	55,598,236	T[25]	MS990	chr11	118,353,038	118,353,053	T[16]
MS470	chr6	117,718,360	117,718,370	T[11]	MS997	chr11	125,490,766	125,490,786	T[21]
MS478	chr6	117,895,423	117,895,436	A[14]	MS1008	chr12	12,024,132	12,024,149	T[18]
MS487	chr6	152,421,908	152,421,922	A[15]	MS1030	chr12	12,032,967	12,032,985	A[19]
MS489	chr6	152,422,170	152,422,186	T[17]	MS1033	chr12	12,036,212	12,036,245	T[34]
MS525	chr7	13,935,862	13,935,873	A[12]	MS1121	chr14	23,652,347	23,652,367	A[21]
MS549	chr7	74,608,741	74,608,753	T[13]	MS1285	chr17	41,256,088	41,256,097	A[10]
MS558	chr7	92,235,952	92,235,963	T[12]	MS1320	chr18	61,873,522	61,873,573	TG[26]
MS569	chr7	116,381,122	116,381,137	T[16]	MS1396	chr22	23,617,095	23,617,118	A[24]
MS581	chr7	140,480,045	140,480,062	T[18]	MS1398	chr22	23,618,595	23,618,609	A[15]

MSIFinder performs microsatellite locus screening on different sequencing panels; therefore, there will be variations in the microsatellite sites for different sequencing panels. For this study, most of the sites were single-nucleotide repeat microsatellite sites. The mononucleotide repeats are believed to be more sensitive and specific for detecting MSI [11, 19]. The repeat length of these loci ranged from 10 to 34 bp, which is consistent with the repeat length of the gold standard loci recommended by the NCI.

### Evaluated performance of MSIFinder

**Fig. 3:**
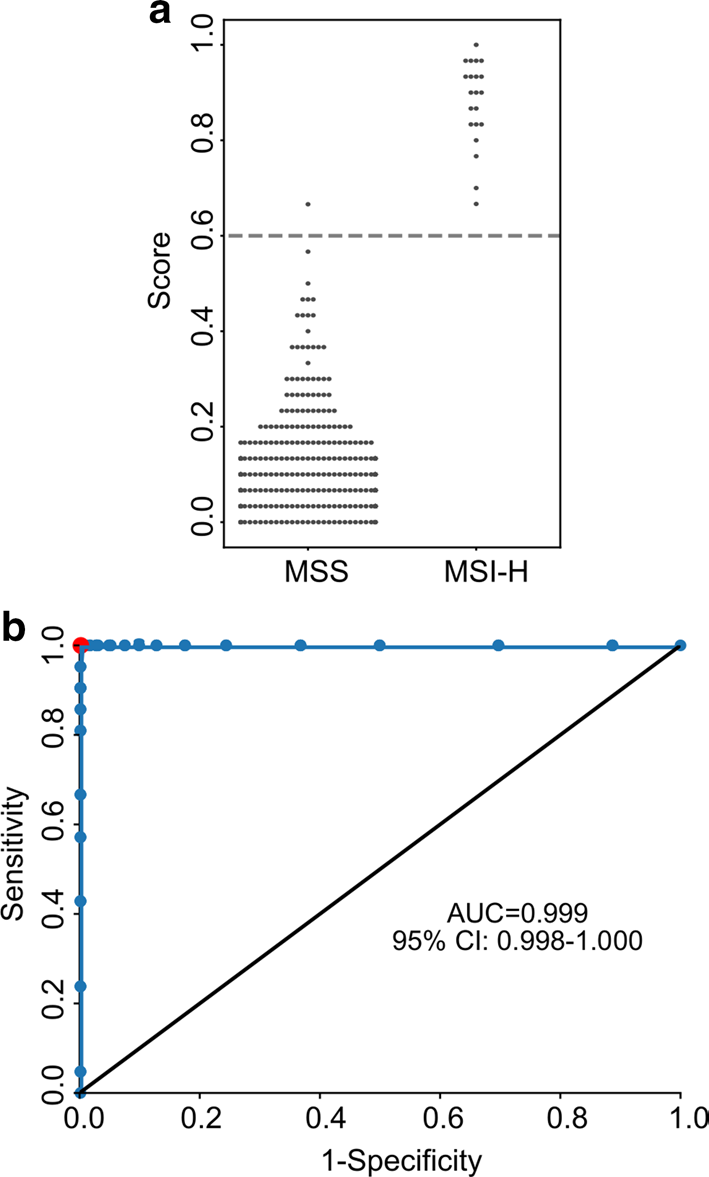
Performance of MSIFinder in the test set. **a** The scatter diagram shows the scores calculated by MSIFinder with 54 microsatellite loci in the test set. Dotted lines represent the threshold. **b** Receiver operating curve (ROC) analysis was used to compare sensitivity and specificity achieved for MSIFinder in the test set

We applied MSIFinder to a test set comprising 21 MSI-H and 379 MSS samples and evaluated its performance by finding the concordances between the status of MSIFinder predicted MSI and MSI-PCR determined MSI. MSIFinder achieved a sensitivity of 1.0, a specificity of 0.997, an accuracy of 0.998, a PPV of 0.954, an F1 score of 0.977, and an AUC of 0.999 (Fig. 3), with only one of the classification errors yielding a false-positive result. These results indicate that MSIFinder can accurately detect MSI status from sample peak data.

### Influence of sequencing depth on the performances of MSIFinder

The performance of software packages that analyze NGS data is affected by the sequencing depth [20]. We processed the peak data to detect whether the sequencing depth affects the performance of MSIFinder. For example, there are two ways to limit the depth of the MSI region to 500× by randomly selecting 500 reads. The first is that if the original depth of the site is greater than 500×, we randomly select 500 reads from this site and then calculate the site peak. The second is that if the original depth of the site is less than 500×, no processing is required.

**Fig. 4:**
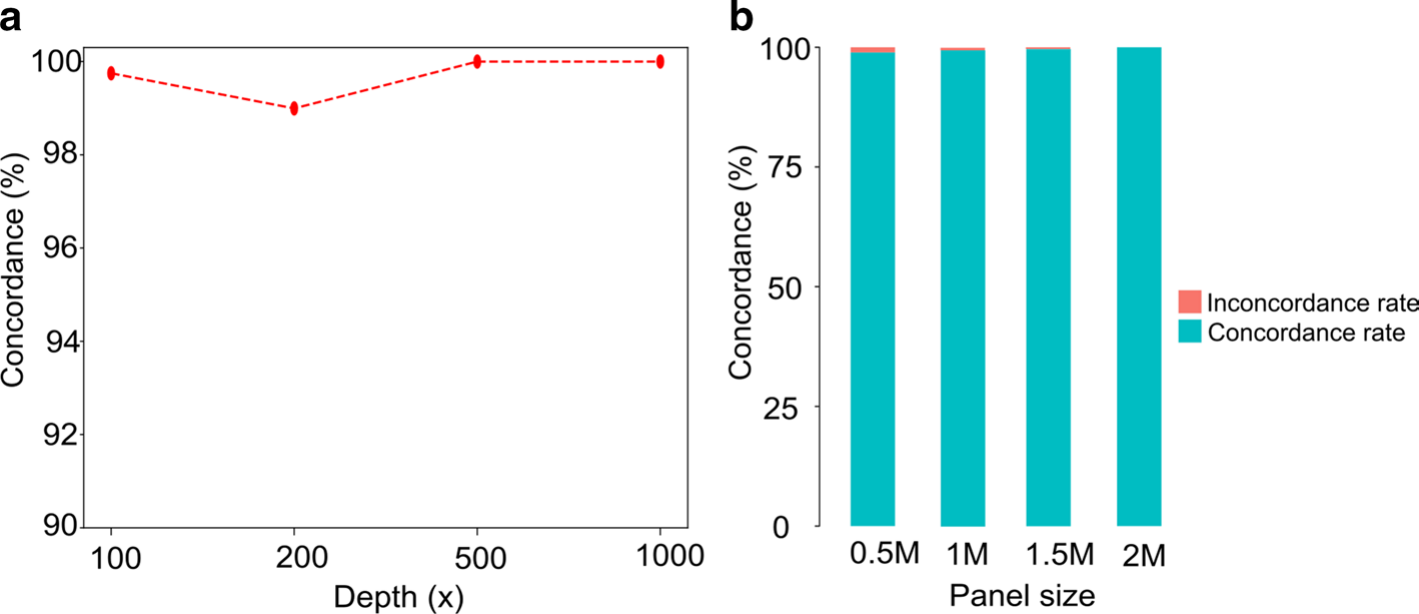
MSIFinder was less affected by the sequencing depth and panel size. **a** The vertical coordinate shows the concordance with different sequencing depths. **b** The y-axis represents the concordance of different panel sizes with a resulting panel size of 2 M. Blue represents the concordance rate, and red represents the non-concordance rate

We observed that when the sequencing depth was reduced to 100×, the concordance was 0.993, and the status of the three samples transformed from MSI-H to MSS. When the sequencing depth was reduced to 200×, the concordance was 0.991, and the status of the two samples transformed from MSS to MSI-H. When the sequencing depth was reduced to 500× and 1000×, the concordance was 1, and the status of the two samples transformed from MSI-H to MSS. (Fig. 4a). For detailed results, see Additional file 1: Table S2. When the sequencing depth was reduced to 1000× or 500×, no false positives or false negatives appeared. With the increase in depth, the sequencing accuracy steadily increases, and even if the sequencing depth is as low as 100× or 200×, the detection accuracy can be maintained above 99%.

### The influence of panel size on the performances of MSIFinder

The inclusion of microsatellite loci that can distinguish between MSI-H and MSS in the sequencing data is influenced by the size of the sequencing panel. To detect the effect of sequencing panel size on MSI detection for MSIFinder, we used random sampling to obtain 100 times 0.5 M panel, 100 times 1 M panel, and 100 times 1.5 M panel, and analyzed the verification samples for each panel.

Notably, the concordance for the 0.5 M, 1 M, and 1.5 M panel was 99%, 99.8%, and 99.9% (Fig. 4b), respectively. For detailed results, see Additional file 1: Table S3. MSIFinder is more robust in terms of the sequencing panel size; even if the sequencing panel is as little as 0.5 M, the error rate is guaranteed to be below 1%.

### Comparison with mSINGS and MSIsensor

**Fig. 5:**
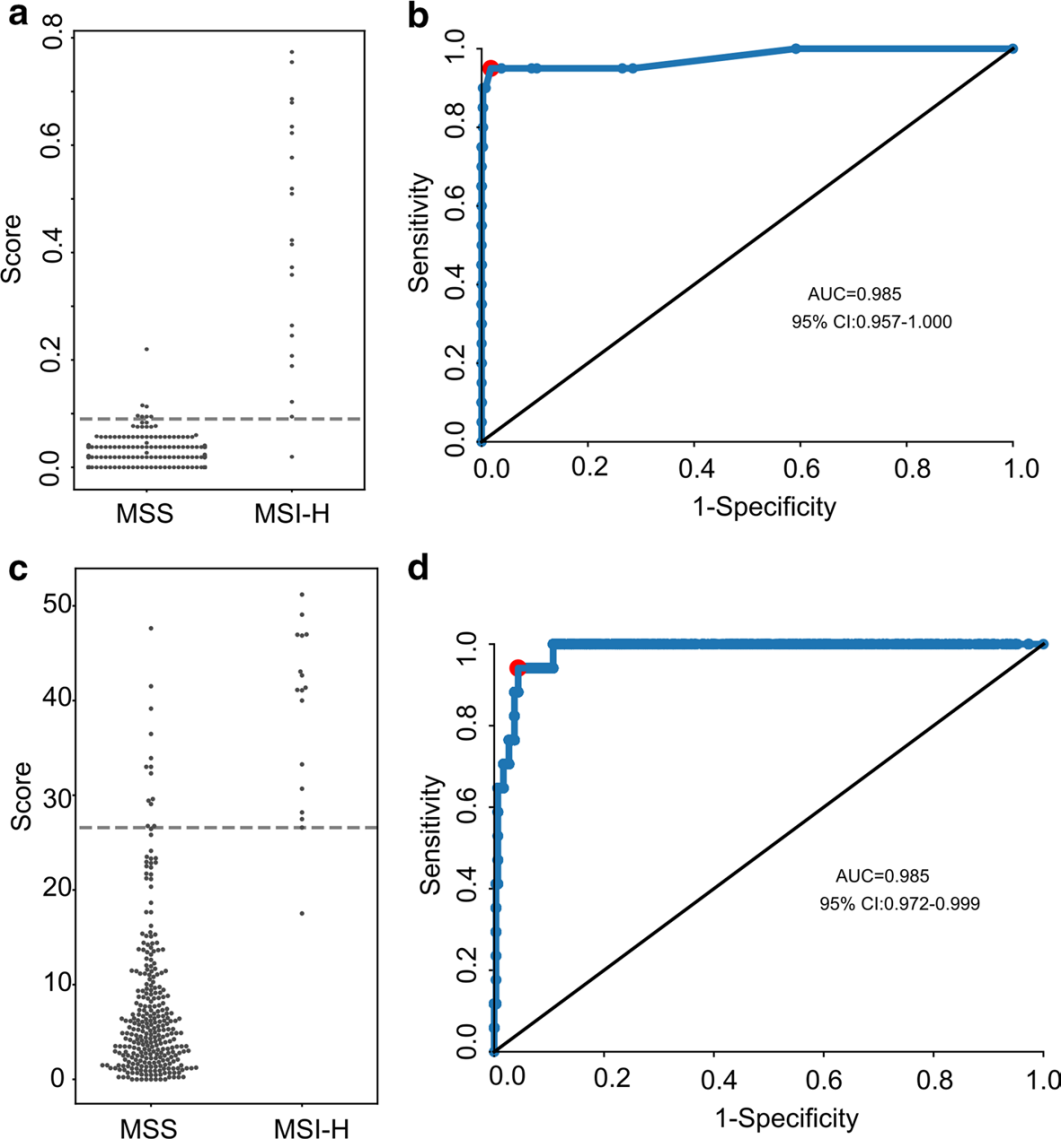
The performance of mSINGS and MSIsensor in the test set. **a**, **c** The scatter diagram shows the scores calculated using mSINGS (**a**) and MSIsensor (**c**) in the test set. Dotted lines represent the threshold. **b**, **d** Receiver Operating Curve (ROC) analysis compares sensitivity and specificity achieved for mSINGS (**b**) and MSIsensor (**d**) in the test set

We compared MSIFinder with two other commonly used MSI detection software packages, mSINGS and MSIsensor. As observed in Fig. 5, for mSINGS, we chose 0.09 as the best cutoff and obtained the highest AUC (0.985). For MSIsensor, we chose 26.58 as the best cutoff and derived the highest AUC (0.985). However, the performance of the software may not be comprehensively evaluated from a single indicator such as the AUC. As a result, we compared other indicators such as sensitivity, specificity, accuracy, and PPV (Table 2). All the indicators of MSIFinder were higher than those of mSINGS and MSIsensor. For detailed results, see Additional file 1: Tables S4–S6.

**Table 2:** Summary of classification performance of MSIFinder, mSINGS and MSIsensor

Tools	Sen	Spe	Acc	PPV	F1	AUC
MSIFinder	0.997	1.000	0.998	0.954	0.977	0.9999
msings	0.983	0.950	0.981	0.730	0.826	0.985
MSIsensor	0.959	0.944	0.958	0.586	0.723	0.985

Sen: sensitivity; Spe: specificity; Acc: accuracy; PPV: positive predictive value; F1: F1 score; AUC: area under curve

### Prospective cohort to verify the robustness and effectiveness of MSIFinder

**Fig. 6:**
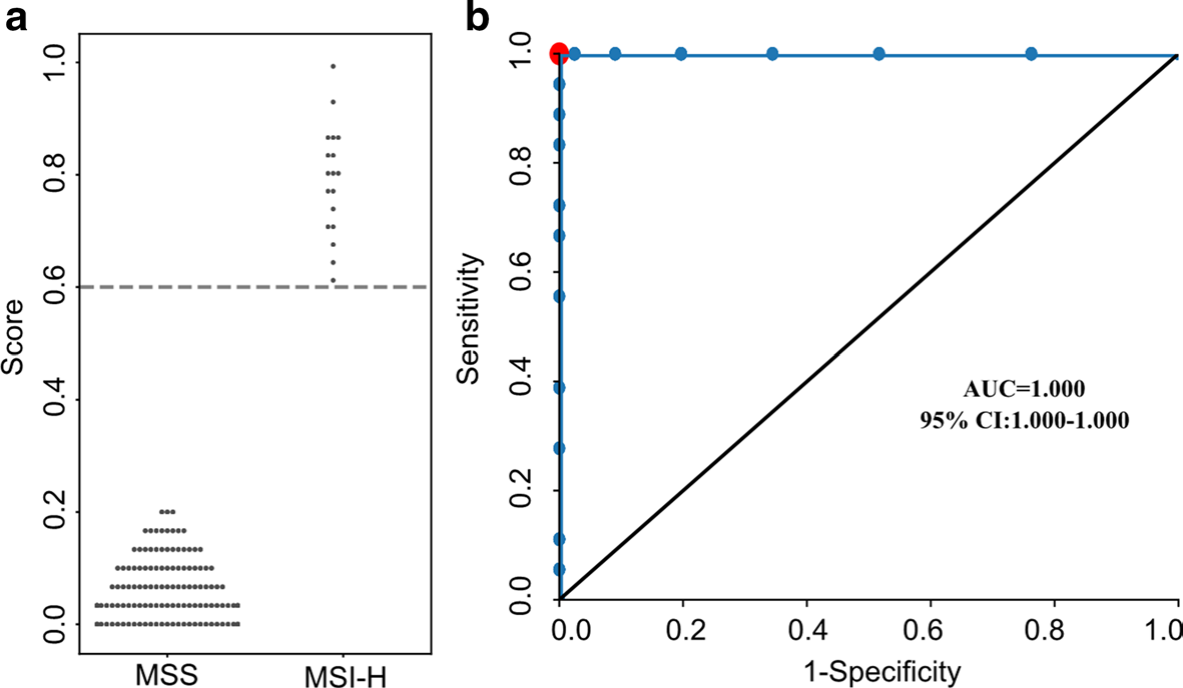
Prospective cohort to verify the robustness and effectiveness of MSIFinder. **a** The scatter diagram shows the scores calculated by MSIFinder with 54 microsatellite loci in a prospective cohort. Dotted lines represent the threshold. **b** Receiver Operating Curve (ROC) analysis was used to compare sensitivity and specificity achieved for MSIFinder in the prospective cohort

As the MSI-H sample number of the testing set was 25, the performance of MSIFinder on the testing set mentioned above might be over-optimistic. To further verify the robustness and effectiveness of MSIFinder, we applied MSIFinder to a prospective cohort consisting of 140 samples. Of 140 tumors, 18 were determined as MSI-H, and the remaining 122 were determined as MSS using MSI-PCR. With this prospective cohort, MSIFinder achieved a sensitivity (recall) of 1.0, a specificity of 1.0, and an AUC of 1.0 (Fig. 6). For detailed results, see Additional file 1: Tables S9. These results indicated that MSIFinder is a robust and effective tool for MSI classification.

## Discussion

We built an RFC classifier based on a training set consisting of 19 MSI-H and 25 MSS samples. The classification performance of MSIFinder was tested by a validation set that included multiple tumor types comprising 21 MSI-H and 379 MSS samples and a prospective cohort consisting of 18 MSI-H samples and 122 MSS samples. MSIFinder was less affected by panel size and sequencing depth. These results suggest that MSIFinder is a robust classification tool with high accuracy, sensitivity, and specificity.

MSIFinder targets only specific sequencing panels that are greater than 0.5 M to ensure that the correct rate is greater than 99%; this expands the application range of MSIFinder. When the sequencing depth was reduced to 500×, only one false negative sample was in the validation set. When the sequencing depth was reduced to 1000×, no false-positive or false negative samples appeared. When the sequencing depth was reduced to 100×, the detection accuracy could be maintained above 99%.

**Fig. 7:**
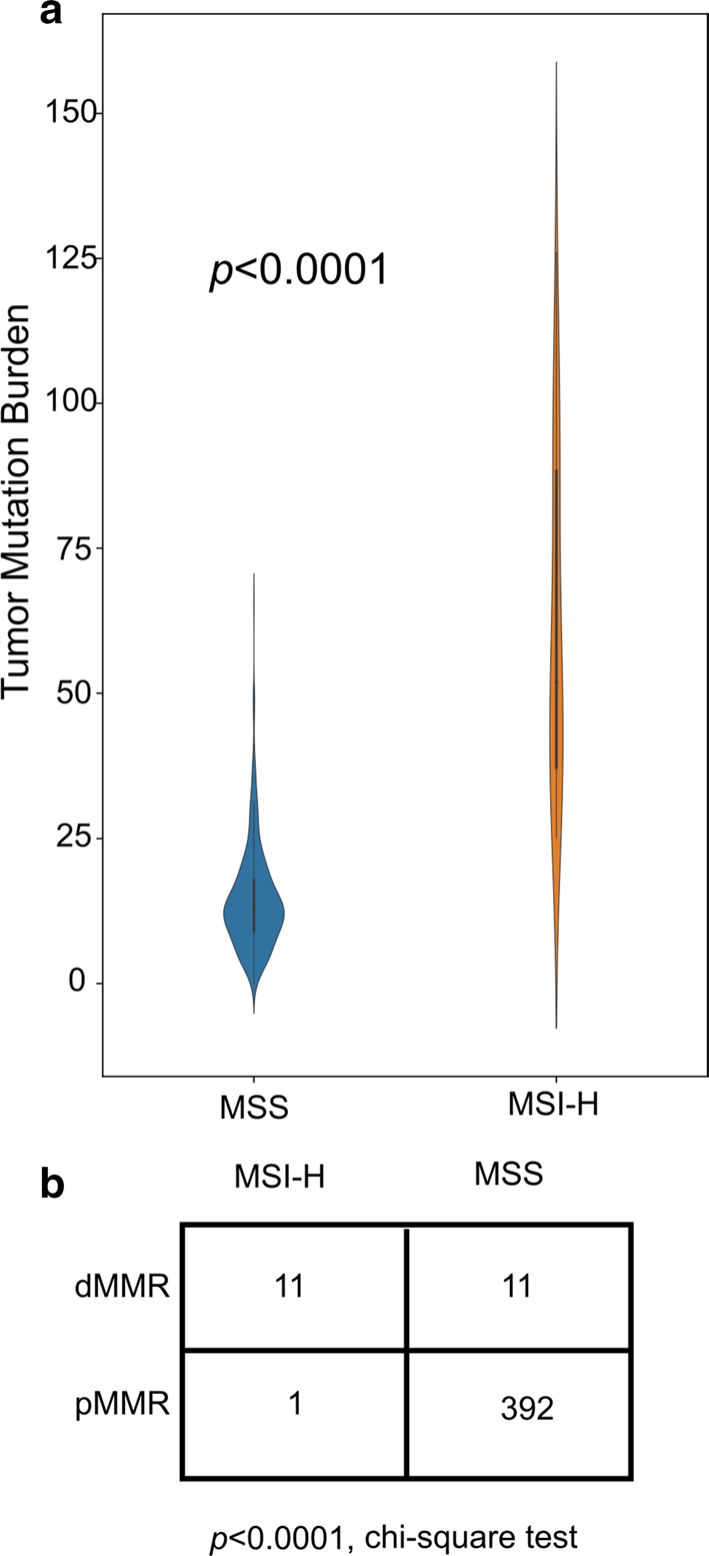
Consistency of TMB and MMR status compared to MSI status

There was a false-positive sample in the validation set. The predicted score of this sample was 0.67, which is greater than the cutoff of MSIFinder (0.6); thus, it was judged to be positive. From Fig. 7a, we observed that the tumor mutation burden (TMB) of MSI-H samples was significantly higher than that of MSS samples. The TMB of this sample was 27.13, which belongs to TMB-H (TMB High); for detailed results, see Additional file 1: Table S7. Some studies have reported that using fewer microsatellite sites in PCR may lead to a missed detection [21–23]. Further, MSIFinder uses 54 sites with specific differences between MSI-H and MSS samples; thus, it is more comprehensive in assessing the microsatellite status of the sample. In this study, we cannot use raw fastq of TCGA or other packages to evaluate the performance of MSIFinder because the downloads need authorization. Therefore, more MSI-H samples will be used for verification in the future, to ensure the accuracy of MSIFinder.

The MSI status of the sample can be inferred from the MMR status. In our sample sets, 419 samples have been MMR tested; thus, we have calculated the correlation between MMR and PCR (Fig. 7b). We used the chi-square test to analyze the consistency of MMR and PCR-MSI (*p* < 0.001). The test revealed a strong correlation between MMR status and MSI status, consistent with existing reports. However, the rate of consistency between the states of MMR and MSI did not reach 80%, indicating that the state of MSI state cannot be completely determined based on the state of MMR.

We compared MSIFinder with two other commonly used MSI detection software packages, MSIsensor and mSINGS. The mechanism of MSIFinder is similar to that of MSIsensor and mSINGS; however, MSIFinder differs in the methods used to select microsatellite sites and determine the MSI status of samples. MSIFinder has two requirements for a selected site. One is that the capture efficiency of the site must be high, and the other is that the site must have high discrimination between MSI-H and MSS samples. To discriminate the sample microsatellite status, MSIFinder uses an RFC classifier. From the results, many indicators of MSIFinder were the highest among three software packages. Apart from MSIFinder, the other software packages are MSIpred [16] and MSIseq [15], which are representative packages obtained via insertions and deletions from the MAF file to predict the state of the sample's MSI status. The MSI status of samples with no loss of function of the mismatch repair gene will be unstable [24].

## Conclusion

In summarize, we propose a software package that detects MSI status in multiple tumor types and is not affected by the panel size and sequencing depth; its accuracy is currently its most important feature.

## Availability and requirements

Project name: MSIFinder.

Project home page: https://github.com/861934367/MSIFinder.

Operating system(s): Platform independent.

Programming language: Python.

Other requirements: Python 3 (version 3.51): pandas (version 0.23.4), pysam (version 0.15.1), matplotlib (version 3.03), seaborn (version 0.9.0) and sklearn (version 0.20.0) packages; external programs: MSIsensor (version 0.6), bedtools (version 2.28.0); BWA (version 0.7.12-r1039), SAMtools (version 0.1.19-96b5f2294a), GATK (version 4.1.0.0), Picard (version 1.72); fastp (version 0.19.3) for reading FASTQ files.

License: GNU GPL, FreeBSD etc.

Any restrictions to use by non-academics: license needed.

## Supplementary information

**Additional file 1: Table S1.** Comparison of the performance of RFC and SVM on the test set. **Table S2**. Detailed information on the samples used in the training set and their score results. **Table S3**. Random depth of detailed result information. **Table S4**. Detailed result information of random panel size. **Table S5**. The detailed information of the sample used in the verification set and its score result. **Table S6**. The detailed information of the sample used by mSINGS and its score result. **Table S7**. The detailed information of the sample used by MSIsensor and its score result. **Table S8**. Sample information used to analyze the relationship between TMB and MSI. **Table S9**. Detailed information of the samples used in the prospective cohort.

**Additional file 2: Fig. S1.** Performance of MSIFinder in the training set. (A) The scatter diagram shows the scores calculated by MSIFinder with 54 microsatellite loci in the training set. Dotted lines represent the threshold. (B) Receiver Operating Curve (ROC) analysis was used to compare sensitivity and specificity achieved for MSIFinder in the training set.

## Data Availability

Not available.

## Abbreviations

AUC: Area under the curve
BAM: Binary SAM files
BWA: Burrows–Wheeler aligner
CRC: Colorectal cancer
GATK: Genomic analysis toolkit
IHC: ImmunoHistoChemistry
MMR: Mismatch repair
MS: Microsatellites
MSI: Microsatellite instability
MSI-H: Microsatellite instability-high
MSI-L: Microsatellite instability-low
MSS: Microsatellite stable
NCI: National Cancer Institute
NGS: Next-generation sequencing
PCR: Polymerase chain reaction
PPV: Positive predictive value
RFC: Random forest classifier
TMB: Tumor mutation burden
TMB-H: Tumor mutation burden high
