# Supplementary figures and images for: MSIFinder: a python package for detecting MSI status using random forest classifier
Source: BMC Bioinformatics. 2021 Apr 12;22:185. doi: 10.1186/s12859-021-03986-z (PMC8042960; doi:10.1186/s12859-021-03986-z)

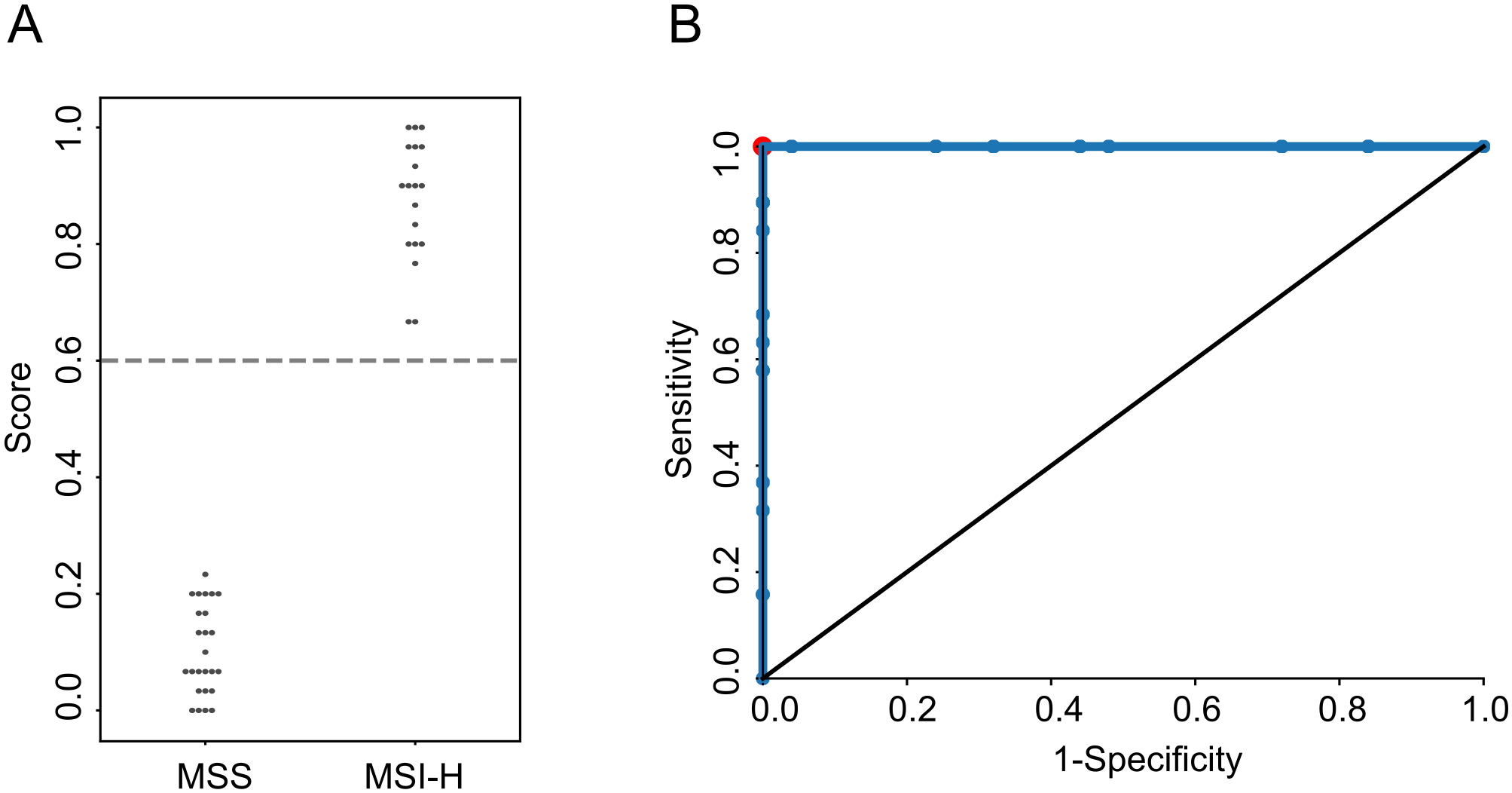

Supplement: Supplementary file 2 — Additional file 2: Fig. S1. Performance of MSIFinder in the training set. (A) The scatter diagram shows the scores calculated by MSIFinder with 54 microsatellite loci in the training set. Dotted lines represent the threshold. (B) Receiver Operating Curve (ROC) analysis was used to compare sensitivity and specificity achieved for MSIFinder in the training set. [file 12859_2021_3986_MOESM2_ESM.tiff]
